# g-force induced giant efficiency of nanoparticles internalization into living cells

**DOI:** 10.1038/srep15160

**Published:** 2015-10-19

**Authors:** Sandra M. Ocampo, Vanessa Rodriguez, Leonor de la Cueva, Gorka Salas, Jose. L. Carrascosa, María Josefa Rodríguez, Noemí García-Romero, Jose Luis F. Cuñado, Julio Camarero, Rodolfo Miranda, Cristobal Belda-Iniesta, Angel Ayuso-Sacido

**Affiliations:** 1 Instituto Madrileño de Estudios Avanzados, IMDEA Nanociencia. Madrid, Spain; 2 Centro Nacional de Biotecnología (CNB-CSIC), Madrid, Spain; 3 Instituto de Medicina Molecular Aplicada (IMMA), School of Medicine, San Pablo-CEU University, Campus de Montepríncipe, Madrid Spain; 4 Departamento de Física de la Materia Condensada, Universidad Autónoma de Madrid & Instituto Nicolás Cabrera, Madrid, Spain; 5 Fundación de Investigación HM Hospitales, Madrid, Spain

## Abstract

Nanotechnology plays an increasingly important role in the biomedical arena. Iron oxide nanoparticles (IONPs)-labelled cells is one of the most promising approaches for a fast and reliable evaluation of grafted cells in both preclinical studies and clinical trials. Current procedures to label living cells with IONPs are based on direct incubation or physical approaches based on magnetic or electrical fields, which always display very low cellular uptake efficiencies. Here we show that *centrifugation-mediated internalization* (CMI) promotes a high uptake of IONPs in glioblastoma tumour cells, just in a few minutes, and via clathrin-independent endocytosis pathway. CMI results in controllable cellular uptake efficiencies at least three orders of magnitude larger than current procedures. Similar trends are found in human mesenchymal stem cells, thereby demonstrating the general feasibility of the methodology, which is easily transferable to any laboratory with great potential for the development of improved biomedical applications.

The possibility of labeling living cells has allowed the development during the last decade of a variety of techniques within the biomedical field, ranging from *in vitro* cell manipulation to imaging-based applications in the clinical settings, especially relevant in cancer and cell therapy^1^,^2^,^3^,^4^. The cell-based therapy relies on the successful delivery of labeled cells into the target site, and therefore, tracking these became a main issue to ensure clinical safety and therapeutic efficacy^5^. Magnetic resonance imaging (MRI) offers several advantages for tracking, such as high resolution, easy accessibility and three-dimensional capabilities^6^,^7^,^8^,^9^. By using iron oxide nanoparticles (IONPs) as contrast agents^10^, it can be done *in vivo* in a non-invasive manner, and it has been shown to be safe and effective. Thus, IONPs-labeled cells is one of the most promising approaches for a quick and reliable evaluation of grafted cells in preclinical studies and clinical trials (Suppl. Table S3)^10^,^11^.

Current labeling procedures, however, present important limitations that undermine its potential, most of them related to the reduced intracellular concentration of IONPs and the time consuming labelling procedure. Most *in vivo* cell-labeling methods with IONPs are based on direct incubation (DI), which relies on standard endocytosis mechanisms and requires long periods of incubation and large IONPs concentrations^12^, because ultimately the cellular internalization of IONPs depends upon the sedimentation and diffusion velocities of the nanoparticles^13^. Large (>100 pg/cell) amounts of internalized IONPs for specific surface coated (charged) nanoparticles have been reported, but using long incubation times (c.a. 24 hours)^14^. Recently, methods using magnetic or electric fields have shown much shorter incubation times (in the scale of minutes), but with much less internalized IONPs (c.a., 10 pg/cell)^15^,^16^,^17^,^18^,^19^,^20^,^21^. Furthermore, in order to achieve the intracellular iron oxide concentrations necessary for MRI, all these methodologies require the use of large initial iron oxide concentrations, ranging from 100 μg Fe/ml up to 2000 mg Fe/ml^22^,^23^,^24^. Taking into account the initial concentration of IONPs, the incubation time, and the resulting internalized amount, all labeling methods up to now present very low uptake efficiencies, <10^−9^ cell^−1^ min^−1^. Finally, existing techniques do not allow controlling the uptake of IONPs into the cells, with the consequent lack of reproducibility among different internalization events, jeopardizing the standardization of the procedure.

Here we introduce a simple and straightforward method of controlled living-cell labeling with IONPs by using centrifugal forces, in a rapid, predictive and quantitative fashion. Our *centrifugation-mediated internalization* (CMI) method allows 100% labeling efficacy with high IONPs internalization (>200 pg/cell) via clathrin-independent endocytosis uptake, in short incubation times (1–20 minutes), and requiring only small initial IONPs concentrations (<50 μg Fe/ml), which results in cellular uptake efficiencies up to 10^−6^ cell^−1^ min^−1^, three orders of magnitude larger than previous ones (Suppl. Table S2). Additionally, consistent with previous reports showing that proteins adsorbed onto particles enhance colloidal stability instead of diminishing it, the concentration of FBS reduces the hydrodynamic diameter of the IONPs used in the present study from 1014 nm (0% FBS) to 357 nm (10% FBS). Although both two conditions can be successfully used for the CMI method, we decided to use 10% FBS in order to facilitate cell maintenance (see Suppl. Fig. S4)^25^.

Figure 1 shows schematically the CMI method. Dispersed IONPs are held in a container with cells pelleted at bottom. Under the influence of centrifugal force, IONPs move through the solution over the cell pellet (see Suppl. video) with steady velocities much larger than those provided by gravity in the DI method (Suppl. Sect. 1). IONPs arrive much faster to the living cells and with a momentum several orders of magnitude larger when centrifugal forces are exploited. This improves both efficacy and efficiency of IONPs internalization into the living cells. The identification of adjustable CMI parameters allows, in addition, overall control of living-cell labeling. The principle is illustrated for glioblastoma tumour cells and extended for human mesenchymal stem cells (hMSCs) to prove its generality.

The internalization efficacy and efficiency of CMI has been evaluated in parallel with DI. Experimental, imaging, and quantification methodologies are detailed in Methods. The complete analysis is presented in the Supplementary Information, including the cytotoxicity assays, which indicate no toxicity.

For this study, U251 cells without (control) and with IONPs at 25 μg/ml concentration were directly incubated (DI) for 24 hours or subjected to centrifugal forces (1500 rpm) for 5 minutes (CMI). For efficiency comparison between DI (5 minutes) and CMI (5 minutes) under the same previous condition see Supplementary Fig. S5. Figure 2A reproduces some representative images after Prussian blue staining experiments which show no IONPs internalization in control cells, while the labeling efficiency is similar in the two methods, reaching almost 100% of the cells (see also Suppl. Fig. S6). The images also show that IONPs are distributed homogeneously inside the cells (always outside from the nucleus), with a larger amount obtained by CMI. Quantification of intracellular iron content by inductively coupled plasma-optical emission spectrometry (ICP-OES) measurements confirm that the total amount of IONPs by CMI is almost 5 times higher, in just 5 minutes, than by DI in 24 hours (Fig. 2B).

In the case of DI, the darker blue areas (as the one marked with a black arrow) could be related with IONPs non-internalized, i.e. attached to cell membrane. Experiments combining scanning electron microscopy (SEM) images with energy dispersive x-ray (EDX) spectroscopy analysis confirm indeed the presence of non-internalized IONPs attached outside the cells. The SEM images of Fig. 2C show cells coated with non-uniform rough-shaped objects, rather abundant in the case of DI (central panel, compare insets). The corresponding EDX compositional analysis indicates that those contain Fe (Fig. 2D). Taking into account the surface sensitivity of the technique, the area below the Fe peak is related to the amount of IONPs mainly outside the cell, which indicates that the amount of non-internalized IONPs is 50 times higher in the case of DI. Note that this observation suggests that reported data on iron concentration per cell by using DI might be grossly overestimated, with direct implications in the evaluation of MRI data. In any case, this clearly demonstrates that IONPs internalization by CMI is significantly more efficient than standard internalization methods based on direct incubation (see Suppl. Table S2).

In order to determine the internalization mechanism of CMI, the time evolution after internalization of IONPs (25 μg Fe/ml, 1500 rpm for 5 minutes) was followed. Figure 3A shows transmission electron microscopy (TEM) images that reveal that 4 hours after internalization IONPs are observed in translucent open vesicles with membranes that are not always closing the whole vesicle perimeter. At longer times, however, the IONPs-containing vesicles evolve towards a typical morphology of early and late endosomes (Fig. 3A, central and lower panels). In all cases, the EDX analysis reveals the presence of iron in these vesicles (Suppl. Fig. S7). These results suggested an endocytosis-independent pathway for the internalization of IONPs using the CMI method. This is in contrast with the receptor-mediated endocytosis mechanism widely demonstrated for standard procedures, including DI^26^,^27^,^28^. To further verify this hypothesis, we examined the presence of receptor-mediated endocytosis by comparing the IONPs uptake, using DI or CMI, in the presence or absence of chlorpromazine (CPZ), an inhibitor of clathrin-dependent endocytosis. Figure 3B–C demonstrates that the inhibitor drastically reduced the uptake of IONPs internalization by DI but not by CMI, indicating that the uptake of IONPs by CMI is independent of the endocytosis pathway. Furthermore, these IONPs remained within the cells for up to 96 hours with a labelling efficiency above 80% (Fig. 3D and Suppl. Fig. S8), qualifying them for medium term cell-labeling applications.

In order to identify the key parameters controlling the CMI efficiency, the influence of frequency of rotation, amount of cells, initial IONPs concentration, and centrifugation time on IONPs internalization has been studied. The efficiency remained unchanged when the amount of U251 cells and the initial IONPs concentration are increased proportionally (see Suppl. Fig. S9). We did observe a slight influence of the frequency of rotation on IONPs internalization (see Suppl. Fig. S10), within tolerable ranges for cells. In contrast, both the initial IONPs concentration ([IONP]_0_) and the centrifugation time (*t*_CF_) affect strongly the internalization efficiency. The images of Fig. 4A correspond to different [IONP]_0_ concentrations (increasing towards the bottom) and centrifugation times, *t*_CF_ (increasing towards the right), while keeping fixed the frequency of rotation (1000 rpm) and the amount of cells (50.000). It is clearly observed that the right-bottom image shows the larger amount of internalised IONPs. For a given centrifugation time, the labelling efficiency increases with increasing initial IONPs concentration, reaching 100% for 50 μg/ml even for the shortest centrifugation times explored (Fig. 4B). Quantitative ICP-OES measurements of the Fe content shows that, once all cells are labelled, the Fe content per cell increases with the square root of centrifugation time *t*_CF_ (Fig. 4C), indicating that it is a diffused limited process (see model in Suppl. Sect. 1). The general trend is summarised in Supplementary Fig. S2. In brief, for a given centrifugation time, the intracellular IONPs content ([IONP]_cell_) is proportional to [IONP]_0_ (see Suppl. Fig. S11A), pointing out that sedimentation is a relevant process, and follows a square-root of time law characteristic of a limited diffusive process. Finally, we have to mention that the internalization caused by the CMI method does not have toxic effects for the cells (Suppl. Fig. S11B).

The method described above can be used for tuning the amount of IONPs internalized into cells in a controlled way and might be extended to any other living cell for different biomedical applications. The general character of our CMI method is illustrated using human mesenchymal stem cells (hMSCs), one of the most utilized cell types in clinical trials for which *in vivo* cell tracking is necessary. The images of Fig. 5 correspond to different [IONP]_0_ concentrations (increasing towards the bottom) and centrifugation times, *t*_CF_ (increasing towards the right), while keeping fixed the frequency of rotation (1000 rpm) and the amount of cells (50.000). The amount of internalised IONPs into hMSCs increases towards the right and the bottom as in the previous experiments with U251 cells. The percentage of labelling correlates with IONPs concentration and centrifugation time, reaching 100% labeling efficiency for concentrations of 75 μg/ml regardless the CF application time. There are no toxic effects detectable for the cells (Suppl. Fig. S12).

In **conclusion**, CMI is currently the most efficient (see Suppl. Table S2), reproducible, affordable and easy procedure to successfully internalize IONPs into living cells. This has been demonstrated with glioblastoma tumour cells and mesenchymal stem cells. Both relevant biological information and performance capability have been addressed. Centrifugation-mediated internalization allows labelling cells with controlled amount of IONPs in few minutes, reducing costs and cell-labeling time, with potential implications in patient hospitalization periods, and further decreasing the possibility of contamination and avoiding biological alterations of cells before grafting. In addition, the required technology is already available in any laboratory and easily transferable to an environment with good manufacturing practice (GMP) conditions. Thus, CMI could have important applications in both preclinical and clinical studies.

## Materials and Methods

### IONPs

IONPs of 12 nm diameters were synthesized and characterized according to the protocols described in supplementary materials. Before cell incubation, IONPs were dispersed by sonication for 5 minutes and filtered through a 0,22 μm filter (Millex-GP, Merck-Millipore Darmstadt, Germany) in presence of medium containing 10% FBS or 10% HS until desired concentration, and finally this mix was sonicated again for 1 minute before incubations.

### Cell culture

U251 glioblastoma cell line was purchased from American Type Culture Collections (Manassas, VA, USA). This cell line was grown as monolayer in Dulbecco’s Modified Eagle’s Medium (DMEM) supplemented with fetal bovine serum (FBS) at a final concentration of 10%, 2 mM L-glutamine, 1 *μ*g/ml fungizone and 100 U/ml of penicillin and 100 μg/ml streptomycin. All the media, serum, L-glutamine, fungizone and antibiotics were purchased from GIBCO. Cell line was maintained at 37 °C in a humidified atmosphere consisting of 75% air and 5% CO_2_ in an incubator. Human mesenchymal stem cells (hMSCs) (a gift from Dr. Carmen Escobedo Lucea) were grown in Dulbecco’s Modified Eagle’s Medium (DMEM) supplemented with human serum (HS) at a final concentration of 10%, 100 U/ml of penicillin and 100 μg/ml streptomycin.

### Internalization of IONPs

#### Direct incubation (DI)

Cells were plated in a 24 well plate at 2,5 × 10^4^ cells per well in 500 μl of DMEM containing 10% FBS. After 24 hours, the growth medium was removed and cells were cultured with IONPs dispersed in fresh medium at a concentration of 10, 25 and 50 μg Fe/ml for 24 hours at 37 °C. A plate of control cells was prepared in a similar manner without the addition of IONPs. For Prussian blue staining, the cells were seeded on 12 mm square glass coverslips (Maienfeld GmbH & Co.KG, Germany) placed into the wells.

#### Centrifugation-mediated internalization (CMI)

U251 glioblastoma cells were initially plated and grown to 80% confluence in 100 mm culture dishes in 10 ml of DMEM containing 10% FBS. The growth medium was aspirated and the cells were washed twice with PBS 1 x (GIBCO), detached with TrypLE^TM^ Express (GIBCO) and resuspended in 1 ml of medium containing 10% FBS. Cell concentration was determined by hemocytometer using Trypan blue dye (GIBCO), and then 5 × 10^4^ cells were placed in tubes of 15 ml and centrifuged at 1000 rpm by 5 minutes. The supernatant fluid was removed and then 500 μl of IONPs at concentrations of 10, 25 and 50 μg Fe/ml were added over the pellets and centrifuged at different times and rates. After that, the medium containing IONPs was exchanged with fresh medium and the cells were resuspended and plated in a 24 well plate. After 24 hours at 37 °C, cell viability and uptake assays were performed and the samples were processed for iron quantification by ICP-OES. A plate of control cells was prepared in a similar manner without the addition of IONPs. For Prussian blue staining, the cells were seeded on 12 mm square glass coverslips (Maienfeld GmbH & Co.KG, Germany) placed into the wells. All centrifugation procedures were carried out with an Eppendorf 5804 using a Eppendorf **A-4**–**44 Rotor** and Buckets (radius = 159 mm), so the conversion between RPM used and G-forces remains as follow 1000 rpm = 178 g; 1250 rpm = 278 g and 1500 rpm = 400 g.

#### Prussian blue staining

After the internalization of IONPs in U251 cells or hMSC by DI or CMI, the cells were washed twice with PBS (AMRESCO, Ohio, USA) and fixed with 4% paraformaldehyde solution for 30 minutes at room temperature. Again, cells were washed twice with PBS, and then were incubated with a 1:1 mixture of 4% potassium ferrocyanide (Sigma-Aldrich) and 4% hydrochloric acid (Sigma-Aldrich) (Prussian blue staining solution) for 15 minutes at room temperature and washed with distilled water three times. The counterstaining was done for cytoplasm with neutral red 0.5% (Panreac Química S.L.U) for 2 minutes at room temperature. After drying the cells, a cover slip was mounted by using the mounting medium DePeX (SERVA Electrophoresis GmbH) and finally, the cells were observed using light microscopy (Leica DMI3000B, Leica Microsystems, Germany). All experiments were carried out in triplicate. A table with the total number of cells considered for all experiments are available in Supplementary Section 2 (Suppl. Table S4).

#### Iron quantification

The iron content in the samples was determined by inductively coupled plasma-optical emission spectrometry (ICP-OES, PerkinElmer Optima 2100 DV ICP) after dissolving the samples in HCl (Fluka Analytical, for trace analysis, ≥37%).), followed by 30 minutes of sonication at 40 °C and diluting them with doubly distilled water.

#### Endocytosis inhibition assay

To elucidate the uptake pathways of IONPs by DI or CMI methods, we used chlorpromazine, a clathrin inhibitor. For the DI method, U251 cells were plated in a 24 well plate at 5 × 10^4^ cells per well and incubated overnight in the growth medium with 10% FBS at 37 °C in 5% CO_2_. 24 hours after that, the growth medium was removed and cells were treated with IONPs dispersed in fresh medium at a concentration of 50 μg Fe/ml alone for 5 hours at 37 °C in 5% CO_2_ or with addition of chlorpromazine hydrochloride (Sigma-Aldrich) at 10 μg/ml. For the CMI method, 5 × 10^4^ cells per tube of 15 ml were divided and centrifuged at 1000 rpm for 5 minutes. The supernatant was removed and then, 500 μl of IONPs at 50 μg Fe/ml were added over the cell pellets and centrifuged at 1500 rpm with or without addition of chlorpromazine hydrochloride at 10 μg/ml. Then, the medium was exchanged with fresh medium alone or with addition of chlorpromazine hydrochloride at 10 μg/ml and cells were plated in a 24 well plate and incubated at 37 °C for 5 hours. A plate of control cells was prepared in a similar way without the addition of IONPs and inhibitor. At the end of the treatment by DI or CMI methods, the medium was removed and cells were washed twice with PBS, fixed with 4% paraformaldehyde for 30 minutes at room temperature, washed again twice with PBS and Prussian blue staining was performed as described before. The experiment was performed in triplicate.

#### Sample processing for Transmission Electron Microscopy (TEM)

For ultrastructural studies, U251 cells adhered to coverslips (both cells alone or cells with IONPs) were fixed in 2% paraformaldehyde and 2.5% glutaraldehyde for 1 hour and processed for visualization at TEM according to the protocol described in the supplementary material.

#### TEM-EDX Analysis

The prepared TEM samples were also analyzed with a scanning transmission electron microscope (STEM, JEOL JEM 2100, Japan) equipped with an energy dispersive x-ray spectrometer (EDX) (INCA Oxford, USA) to determine the local composition of target areas at the nanoscale level. The TEM images were taken at an acceleration voltage of 200 kV with point resolution of 0.25 nm. Energy dispersive spectra analysis was carried out to identify the element composition of selected areas.

#### Statistical analysis

All the data obtained were plotted and statistically analyzed using the software package GraphPad Prism version 5.0 for Windows. All samples were compared using a one-way ANOVA and Bonferroni post-hoc test (*P < 0.05, **P < 0.01, and ***P < 0.001). Only significant differences among the samples are indicated in the charts.

## Additional Information

**How to cite this article**: Ocampo, S. M. *et al.* g-force induced giant efficiency of nanoparticles internalization into living cells. *Sci. Rep.* **5**, 15160; doi: 10.1038/srep15160 (2015).

## Supplementary Material

Supplementary video

Supplementary Information

## Figures and Tables

**Figure 1:**
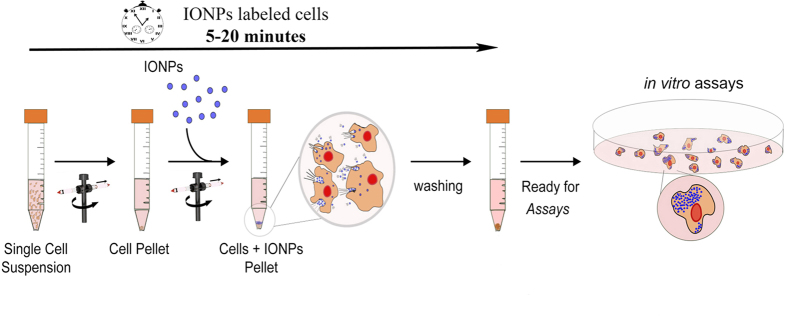
Centrifugation Mediated Internalization (CMI) of Iron Oxide NanoParticles (IONPs) into living cells. Scheme depicting the different steps of the CMI method for IONPs internalization into living cells (See also Supplementary Video).

**Figure 2:**
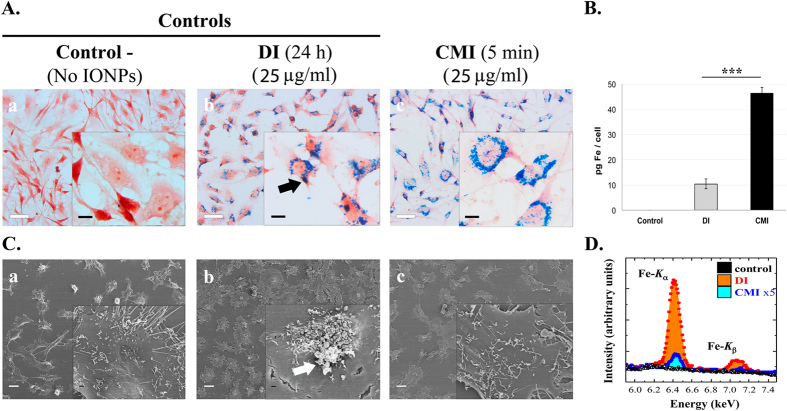
Increased efficacy and efficiency of IONPs internalization into living cells by CMI. (**A**) Representative Optical Microscopy images of Prussian blue staining of control cells without IONPs (left panel); cells incubated for 24 hours with IONPs at 25 μg Fe/ml by the DI method (central panel); and cells incubated for 5 minutes with IONPs at 25 μg Fe/ml and internalized by CMI method at 1500 rpm (right panel). DI produces IONPs aggregation on the cell surface (see the black arrow in the central inset). Scale bar: 40 μm in the main images and 10 μm in the insets. (**B**) Inductively-Coupled Plasma Optical Emission Spectroscopy (ICP-OES) quantification of intracellular iron content of cells incubated with IONPs at 25 μg Fe/ml internalized by DI and CMI methods. **C)** Representative Scanning Electron Microscopy (SEM) images of cells incubated with IONPs at 25 μg Fe/ml by DI and CMI methods. Control cells without IONPs (left panel), cells with IONPs incubated by DI for 24 hours (central panel) and cells with IONPs internalized by CMI at 1500 rpm for 5 minutes (right panel). The white arrow shows the accumulation of IONPs on the cell surface in the DI case. (**E**) Representative Energy Dispersive X-Ray (EDX) spectra analysis reflecting the presence of iron on the extracellular membrane of U251 cells after IONP internalization by DI and CMI methods. Notice the different scales for DI and CMI methods.

**Figure 3:**
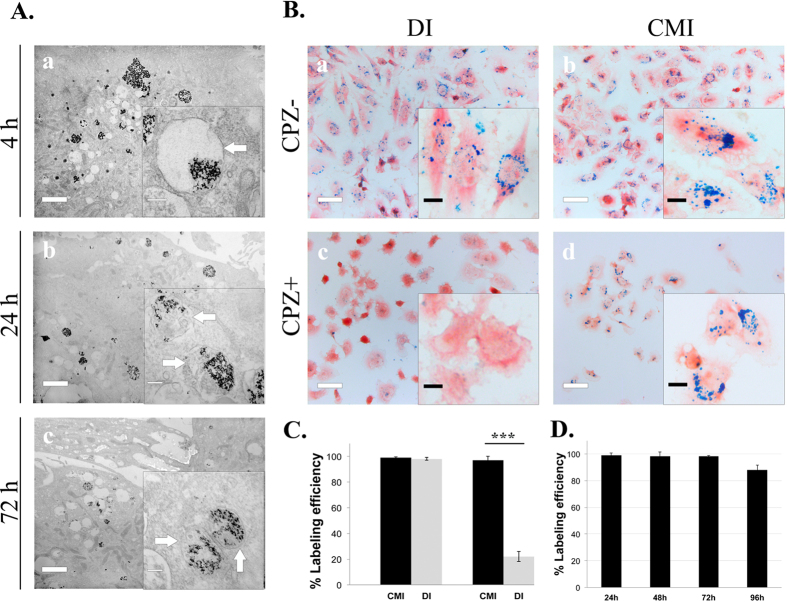
Endocytosis-independent internalization of IONPs into living cells by the CMI method. (**A**) Transmission Electron Microscopy (TEM) images of the time evolution of IONPs internalized by CMI into U251 cells. Scale bar 100 nm in the main images and 20 nm in the insets. (**B**) Endocytosis inhibition by chlorpromazine (CPZ). Prussian blue staining of cells with IONPs (50 μg/ml) internalized by DI (24 hours) or CMI (at 1500 rpm for 5 minutes) in the presence or absence of the clathrin-inhibitor by chlorpromazine (CPZ) (10 μg/ml) for 5 hours. Data are representative of 3 separated experiments. The blue stain density reflects the level of IONPs accumulation within cells. Scale bar: 40 μm in the main images and 10 μm in the insets; (**C**) Labelling efficiency for both methods (as given by the percentage of Prussian blue positive cells) without and with the clathrin-inhibitor CPZ; (**D**) Labelling efficiency of IONPs as a function of time after internalization. Note that even after 96 hours the efficiency is larger than 80%. Error bars represent the standard deviation. ***P < 0.001.

**Figure 4:**
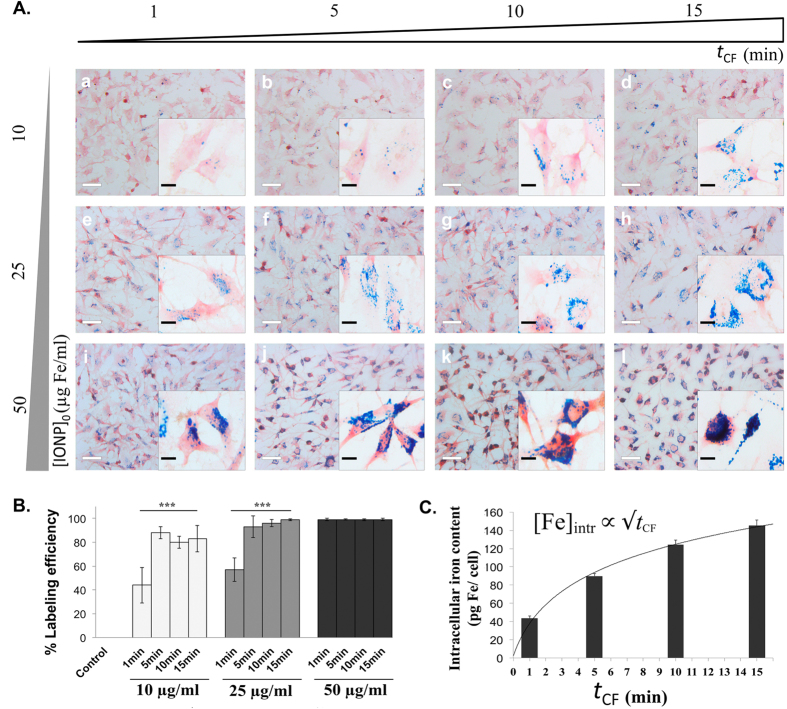
The extent of the IONPs internalization by CMI is proportional to the initial IONPs concentration and the centrifugation time. (**A**) Optical microscopy images of U251 cells after Prussian blue staining following IONPs internalization by CMI at 1000 rpm and different IONPs concentrations (10, 25 and 50 μg/ml) and Centrifugal Force application times (1, 5, 10 and 15 minutes). Scale bar: 40 μm and 10 μm; (**B**) Labeling efficiency of U251 with IONPs internalized by CMI in all different sceneries. (**C**) Intracellular iron content per U251 cell measured by ICP as a function of centrifugation time for an initial Fe concentration of 50 μg/ml. Error bars represent the standard deviation ***P < 0.001.

**Figure 5:**
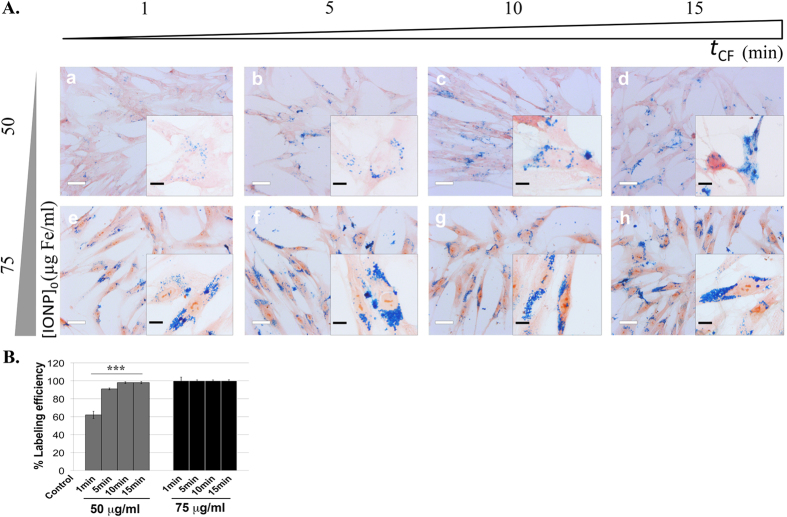
IONPs internalization by CMI is applicable to hMSCs. (**A**) Optical microscopy images of hMSCs cells after Prussian blue staining following IONPs internalization (50–75 μg/ml) by CMI at 1000 rpm and different CF-application time (1, 5, 10 and 15 minutes); (**B**) Labelling efficiency of hMSCs with IONPs internalized by CMI in all different sceneries. Scale bar: 40 μm, in the main images, and 10 μm, in the inserts.
