# Erratum: g-force induced giant efficiency of nanoparticles internalization into living cells

**DOI:** 10.1038/srep17246

**Published:** 2016-01-07

**Authors:** Sandra M. Ocampo, Vanessa Rodriguez, Leonor de la Cueva, Gorka Salas, Jose L. Carrascosa, María Josefa Rodríguez, Noemí García-Romero, Jose Luis F. Cuñado, Julio Camarero, Rodolfo Miranda, Cristobal Belda-Iniesta, Angel Ayuso-Sacido

Scientific Reports 5: Article number: 1516010.1038/srep15160; published online: 10 19 2015; updated: 01 07 2016.

The original version of this Article contained a typographical error in the spelling of the author Jose Luis F. Cuñado, which was incorrectly given as Jose Luis, F. Cuñado.

In addition, there were errors in the Author Contributions.

“S.M.O., V.R. and A.A.S. wrote the main manuscript text. S.M.O. and V.R. carried out experimental works. S.M.O., V.R. and A.A.S. made Main Figures and supplementary figures and tables, except for those mentioned below. GS made supplementary figure 1. S.M.O., J.L.F.C. and J.C. made supplementary table 2. J.L.F.C., J.C. and A.A.S. wrote the supplementary section 1 (Model). L.C. and G.S. produced the nanoparticles. M.J.R. and J.L.C. took the E.M. pictures. S.M.O., N.G.R., J.C., R.M., C.B.I. and A.A.S. reviewed the manuscript. Artworks All the artworks within the main and supplementary figures were created by the authors ad hoc for this manuscript.”

now reads:

S.M.O., V.R. and A.A.S. conceived and designed the experiment with assistance from J.C. L.C. and G.S. synthesized and characterized the IONPs. S.M.O., V.R. and A.A.S. conducted the cell culture assays, including the prussian blue staining optical microscopy and SEM-EDX analysis. J.L.C. and M.J.R. carried out the TEM microscopy analysis. J.L.F.C., J.C. and R.M. performed the IONPs’ living-cell uptake kinetic model of the DI and CMI methods. All authors contributed to the general discussion and comment on the manuscript. S.M.O., V.R. and A.A.S. wrote the manuscript with inputs form J.C., R.M. and C.B.-I.

These errors have now been corrected in the PDF and HTML versions of the Article.

