# Supplementary material for: g-force induced giant efficiency of nanoparticles internalization into living cells
Source: Sci Rep. 2015 Oct 19;5:15160. doi: 10.1038/srep15160 (PMC4609925; doi:10.1038/srep15160)
Supplement: Supplementary Information [file srep15160-s2.pdf]

SUPLEMENTARY INFORMATION

g-force induced giant efficiency of nanoparticles internalization into living cells.

Sandra M. Ocampo, Vanessa Rodriguez, Gorka Salas, Leonor de la Cueva, Jose. L. Carrascosa, María Josefa Rodríguez, Noemí García-Romero, Jose Luis F. Cuñado, Julio Camarero, Rodolfo Miranda, Cristobal Belda-Iniesta and Angel Ayuso-Sacido.

SUPPLEMENTARY SECTION 1

Model

In standard liquid-based cell culture incubation systems, the amount of particles associated with cells at any time is a function of the rate of delivery of particles to the cells, the rates of cellular uptake and loss by degradation or exocytosis.[1] The latter involves biological activity between internalized particles and cell medium and, in principle, it can be considered similar for both DI and CMI methods. The rate of delivery is related with the transport of particles in a liquid and the cellular uptake rate depends on the settling velocity. In this context, three primary processes transport nanoparticles in static (non-flowing) uniform solutions comprising the majority of liquid-containing cell culture systems: diffusion, sedimentation, and advection (transfer by motion of the fluid).[2] Advective forces in cell culture medium held at constant temperature, without disturbance, are minimal and do not significantly affect particle transport. Thermal motion manifests itself on the microscopic scale in the form of Brownian motion, resulting from their collision with the quick atoms or molecules in the liquid, and on the macroscopic scale in the form of diffusion. Gravitational fields, either gravity or centrifugal force, provide the driving force in sedimentation.

In current living-cell labelling procedures, cells are immobilized at the bottom of a culture plate, and incubated with a suspension of nanoparticles. The nanoparticles are assumed to be well-dispersed in the culture medium as a result of diffusion or Brownian motion, so the concentration of nanoparticles at the cell surface is assumed to be the same as that of the initial bulk concentration. However, particles immersed in a liquid also experience a motion originated from a steady gravitational drift, causing the dose of nanoparticles on the cell surface to vary.[3] In such cases, the actual concentration of nanoparticles for cellular uptake could be significantly different from the initial value.

In the following, first it is discussed an important aspect about sedimentation in order to understand the differences between DI and CMI methods. Second, from the diffusion equation, the main experimental observations are reproduced.

Sedimentation aspects: settling velocity

Settling is the process by which particles settle to the bottom of a liquid. In the manuscript are compared two sedimentation procedures for labelling living cells with IONPs:

- Sedimentation by gravity (*direct incubation* DI), the most commonly used incubation procedure,
- Sedimentation by centrifugation (*centrifugation-mediated-internalization* CMI).

Under the influence of either gravity (DI) or centrifugal force (CMI), SPIONs move thorough the solution over the cell pellet (see **Suppl. Fig. 1A**). The effective force acting on a given particle in a solution is given by the competition between the gravitational force F_G (weight or centrifugal) and the force due to buoyancy F_B (weight of displaced fluid), and the force due to viscous drag F_D. For small particles moving not too rapidly, accordingly to the laminar flow Stokes' law, the latter is directly proportional to the sedimentation velocity of the particle $v_P(t)$, i.e., $F_D = f\,v_P$, where in general f is a friction parameter that depend on the liquid properties and on the particle geometry. As soon as the particle starts to move, the drag will come into operation opposing the motion. As the particle accelerates the drag increases, until this drag force just balances the difference of the gravitational and buoyancy. The sedimentation movement equation can be written as a function of this velocity:

$$m_P \frac{dv_P}{dt} = F_G - F_G - f v_P \text{ , (S1)}$$

and its solution is

$$v_P(t) = \frac{F_G\ - F_B}{f}\left(1 - e^{-t\frac{f}{m_P}}\right) \text{ (S2)}$$

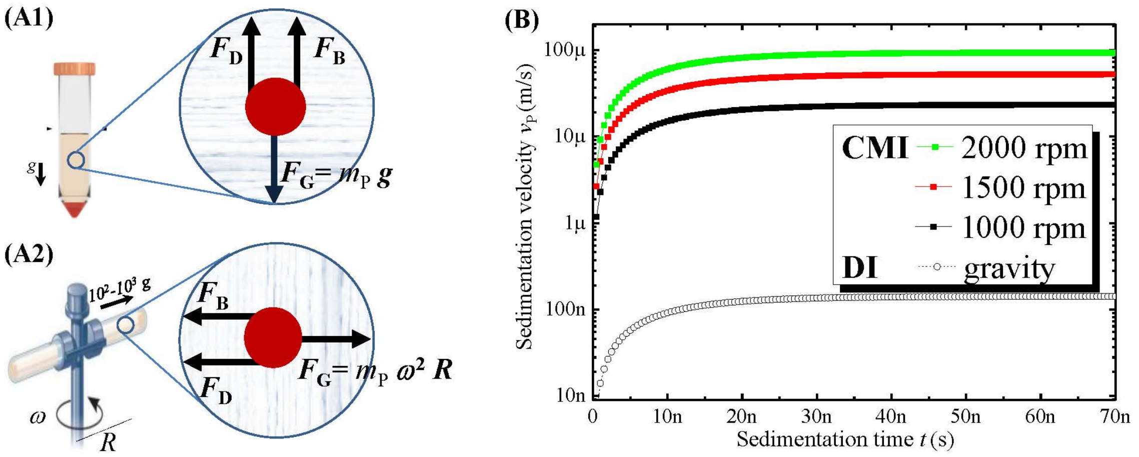

SFig1. Forces and sedimentation velocities under gravitational fields. Schematic sketch of a particle in solution driven by gravity **(A1)** and centrifugal **(A2)** forces. To a particle both DI and CMI methods are qualitatively indistinguishable; however, its intensity may be orders of magnitude greater for the CMI case. **(B)** Kinetics of sedimentation velocity derived from Eq. S1 using the experimental parameters given in Table S1. Note that the steady regime takes place beyond a relatively low time (< 10 ns) for all cases. By comparison with the DI method, the settling velocities provided by the CMI method are between two and three order of magnitude larger.

Assuming spherical particles (density ρ_P and hydrodynamic radius R_H) dispersed in the culture medium (density ρ_L and dynamic viscosity μ_L) and obeying the Stoke's law, i.e., $f = 6\pi\mu_L R_H$, the steady sedimentation velocity, i.e., the settling velocity, reads:

$$v_P = \frac{2\,R_H^2\,(\rho_P - \rho_L)}{9\mu_L} a_G \quad \text{(S3)},$$

where a_G is the acceleration due to gravity and centrifugal force for DI and CMI methods, respectively. Therefore, to a particle the two fields are qualitatively indistinguishable, and one may usefully compare the behavior of particles in centrifuges to those just under the gravity field. The large difference between the two methods originates from the force applied to the particle, which results with settling velocity values much larger for the CMI method, as **Supplementary Figure 1B** shows.

The ratio of the force acting on a particle by centrifugation to the force acting on the particle by gravity is usually quoted in terms of relative centrifugal force RCF (RCF= $F_C/F_g = \omega^2 R/g$). However RCF in units of g is usually rearranged to the following form RCF= 1.119 x 10^{-3} (rpm)^2 R, with R in meters. For the experimental conditions explore in this work, RCF varies from 160g to 650g, for R= 15 cm and 1000 rpm and 2000 rpm, respectively. Therefore, SPIONs arrive to the living cells with a settling velocity up to

three orders of magnitude higher when centrifugal forces are exploited. This improves largely both efficiency and rate of the SPIONs' cell uptake.

	R_H [nm]	ρ_P [Kg/m^3]	ρ_L [Kg/m^3]	μ_L [Pa.s]	ω [rpm]	R [m]	v_P [μm/s]	v_P / v_{DI}
**DI**	150	5200	1000	0.001			0.14	***1***
**CMI**	150	5200	1000	0.001	1000	*0.15*	22	***163***
					1500		49	***366***
					2000		87	***650***

Table S1. Physical parameters. R_H corresponds with the hydrodynamic radius in culture medium. The settling sedimentation velocity v_P is derived from Eq.S3.

Diffussion: uptake kinetics

The main effect of the CMI method is that the concentration of nanoparticles on the cell surface at a given time is much higher as compared with the DI method, even it may be higher than the initial bulk concentration. In most real situations the concentration profile and the concentration gradient are changing with time, i.e., $n(x,t)$. Fick's second law predicts how diffusion causes the concentration of non-interacting nanoparticles to change with time. It is a partial differential equation which in one dimension reads:

$$\frac{\partial n}{\partial t} = D\frac{\partial^2 n}{\partial x^2} \text{ (S4)},$$

where n is the particle concentration, t and x are the time and distance dimensions, and D is the diffusivity. The Stokes-Einstein equation describes the rate of diffusion as a function of the friction parameter f and temperature (T):

$$D = \frac{k_B T}{f} \text{ (S5)},$$

where k_B is the Boltzman constant. Assuming that D is constant, independent of time and initial concentration, the solution of the differential equation S5 within the thin film approximation gives:

$$n(x,t) = \frac{m_0}{\sqrt{4\pi D t}} e^{-\frac{x^2}{4Dt}} \text{ (S6)},$$

where D (~$10^{-12} m^2 s^{-1}$) is the composition-independent diffusivity, x is the distance from the original interface, and m_0 is the "source" of diffusing species per unit area, which is related to the sedimentation process. In the present case, the exponential is approximately the unit for relatively short times (seconds), e.g., just in one minute the

diffusion length $\sqrt{4Dt}$ of about 20 μm, which is one order of magnitude larger than typical cell size (micrometer scale). Therefore, the concentration evolution reads.

$$n(0,t) = \frac{m_0}{\sqrt{4\pi Dt}} \quad \text{(S7)},$$

which decreases linearly with the square-root of time.

The kinetic behavior of the nanoparticle content internalized into the cell can be estimated directly integrating this concentration with respect to the time,

$$[IONP]_{cell} = \int_0^t n(t)\,dt \;\; \propto \;\; \sqrt{t} \quad \text{(S8)},$$

This linear square-root of time dependence behavior is a general trend in different scientific areas, including physics, biology, geology, and economy, to describe diffuse-limited time evolutions of a set of independent particles.

Comparison with experimental data ∝

In the present work, the IONPs' living-cell uptake kinetics by CMI has been evaluated experimentally by performing different assays as a function of incubation time (t_{CF}), centrifugation frequency (f_{CF}), initial concentration ($[IONP]_0$) and number of cells (N_{cell}). The efficiency trends can be easily identified from the images shown in the manuscript and the Supplementary figures. Qualitatively, the efficiency remained unchanged when the amount of cells and the initial concentration are increased proportionally, i.e., SPIONs are equally distributed into the cells (see **Supp. Fig. 9**). In contrast, the efficiency increases when initial concentration (**Fig.4**, **Fig.5**, **Suppl. Fig. 9**, **Suppl. Fig. 10**), frequency (**Suppl. Fig. 10**), and/or centrifugation time (**Fig. 4** and **Fig.5**) increases.

The iron content per cell $[IONP]_{cell}$ of the different assays was quantified by ICP-OS measurements. **Supplementary Figure S2** summarizes the quantitative analysis, providing the dependence of the $[IONP]_{cell}$ with the aforementioned parameters:

$$[IONP]_{cell} \propto \frac{[IONP]_0}{N_{cell}} \sqrt{f_{CF}\, t_{CF}} \quad \text{(S9)}.$$

The linear square-root of time dependence of the IONP concentration inside the living cells found experimentally is in accordance with the kinetic diffusion model (see **Fig.4C**), which indicates that the uptake is limited by diffusion. In addition, the dependences with initial concentration and centrifugation frequency also indicate that the sedimentation is also a relevant parameter. With respect to the dependence of

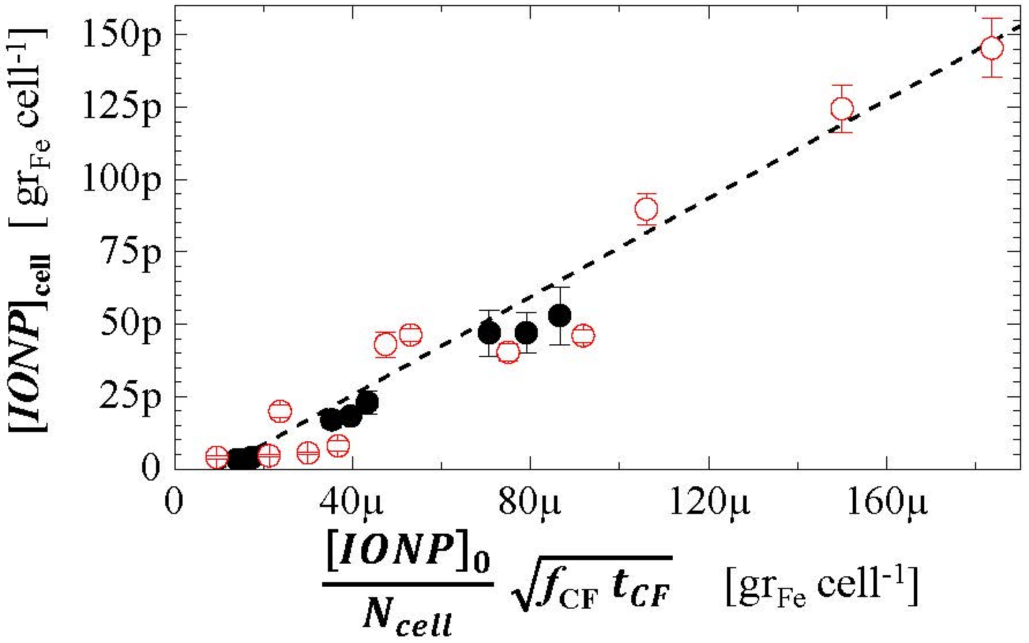

SFig2. Parameters controlling the CMI efficiency. Empty and solid symbols are the intracellular IONPs content $[IONP]_{cell}$ quantified by ICP-OS analysis from the assays presented in Fig.4 and Fig.S7, respectively. The data correspond to different initial concentrations $[IONP]_0$, centrifugation times t_{CF}, and/or centrifugation frequencies f_{CF}. The x-axis has been chosen in order to show all data in one plot. The discontinuous line is a guide for the eye.

$[IONP]_{cell}$ with f_{CF}, it is necessary to mention that the available data are not enough to determine it properly. We have not enough data to determine the corresponding dependences with the conventional DI method, although the trends are expected to be similar but with a much less uptake efficiency.

In summary, in one side sedimentation techniques where the settling force is gravitational in nature are based upon Stokes' law which relates the particle steady velocity to the physical characteristics of the particle-fluid system in which it is settling. So, a much larger amount of particles and high density gradient is close to the targeted cells by using the CMI method. On the other side, the internalization is diffused limited and the cellular uptake kinetics follows a square-root of time, in agreement with the experimental observations.

Finally, in order to evaluate the CMI method, in TableS3 are compared the values of the labelling efficiency, total amount of $[IONP]_{cell}$, uptake rate χ ($\propto = \frac{[IONP]_{cell}}{incubation\ time}$) and the corresponding uptake efficiency ξ_{uptake} ($\xi_{uptake} = \chi\, /\, [IONP]_0$) of selected CMI assays

presented in this work with the records values found by others by using the conventional DI method and/or other physical methods (**Suppl. Table 1)**. The parameters used for the different assays are also indicated. It has to be noted that the CMI method presents 100% efficiency with a very high $[IONP]_{cell}$ value (> 100 pg_{Fe}/cell) in just 5 min. Remarkable, the uptake efficiencies by using CMI are several orders of magnitude larger (> 10^{-7} $cell^{-1}$ min^{-1}) than the corresponding ones by using DI (< 10^{-10} $cell^{-1}$ min^{-1}) and other means (< 10^{-8} $cell^{-1}$ min^{-1}).

Supplementary Table S2: List of representative nanoparticles NPs internalization methods into living cells. The information on NP type, cell type, incubation time, initial NPs' concentration $[NP]_0$, internalized NPs' concentration $[NP]_{cell,}$ are the ones given in the corresponding references. The uptake rate χ and the uptake efficiency have been derived as described in the text.

Method	NP type	Cell type	Incubation time	$[NP]_0$	Labelling efficiency	$[NP]_{cell}$	Uptake rate χ	Uptake efficiency ξ_{uptake}	Ref
				(μg/ml)	(%)	(pg/cell)	(pg/cell min)	($cell^{-1}$ min^{-1})	
DI	IONP	ADSCs	24 h	100	72	25	0,02	1,7E-10	4
DI+PS	Endorem	PBMCs	120 min	100	nd	17±15	0,14	1,4E-09	5
DI + lipofectamine2000	Resovist	hBOECs	24 h	1000	nd	147±6	0,10	1,0E-10	6
Ultrasound	IONP	H22	1 min	410	70	10±2	10,4	2,5E-08	7
Magneto electroporation	Feridex	rMSCs	1 min	2000	nd	11±3	11,0	5,5E-09	8
DI+ NdFeB magnet	IONP	SK-MEL-28	24 h+ 1.5 h	100	nd	204±26	0,14	1,4E-09	9
DI	IONP	U251	24 h	50					This work
CMI	IONP	U251	**1 min**	**50**	**100**	43±4	***43,0***	8,6E-07	
CMI	IONP	U251	5 min	50	100	90±5	18,0	3,6E-07	
CMI	IONP	U251	10 min	50	100	124±8	12,4	2,5E-07	
CMI	IONP	U251	**15 min**	**50**	**100**	**145±10**	9,7	1,9E-07	

SUPPLEMENTARY SECTION 2

Supplementary Methods

Synthesis of IONPs

Iron(III) chloride hexahydrate (≥ 98%, Sigma-Aldrich), sodium oleate (≥ 82%, Riedel-de Haën), oleic acid (90%, Aldrich), meso-2,3-dimercaptosuccinic acid (DMSA, 98% Aldrich), 1-octadecene (90%, Aldrich), n-hexane (99%, Scharlau), toluene (99.8%, Sigma-Aldrich), dimethyl sulfoxide (> 99.5%, Sigma-Aldrich), ethanol (96%, Panreac) were used as received. Dialysis tubing cellulose membrane was purchased from Sigma and washed prior to use. Synthesis of the iron oleate precursor has been described elsewhere[10, 11]. Briefly, the reaction was carried out under nitrogen atmosphere in a round-bottomed flask of 0.5 L, equipped with a mechanical stirrer (PTFE centrifugal stirrer shaft, Ø = 50 mm) thermometer, and entry for nitrogen flow and reflux condenser. A mixture of iron(III) oleate (4.5 g, 5 mmol) and oleic acid (7.5 mmol) in 1-octadecene (50 mL) was heated to 300°C at 8°C/min, and kept at that temperature for 1 hour. The reaction mixture was mechanically stirred only until T = 60°C. The resulting black dispersion was allowed to cool down to room temperature and washed several times with ethanol (10 x 40 ml) centrifugating (RCF = 9000) and with the help of a permanent magnet to separate the magnetic nanoparticles. Finally, ethanol was removed resulting in a black residue of hydrophobic nanoparticles of magnetite that was redispersed in hexane for storing. The iron concentration of the dispersion was measured by Inductively Coupled Plasma Optical Emission Spectrometry (ICP-OES).

The hydrophobic nanoparticles were subjected to surface modification, through ligand substitution with DMSA, for their further dispersion in water[11]. A volume of the stock hexane dispersion containing 50 mg (0.2 mmol) of Fe_3O_4 was washed with ethanol (10 x 10 ml), precipitating the nanoparticles as a black residue that was dried at air. It was redispersed in toluene (20 ml) and added to a solution of DMSA (90 mg, 0.5 mmol) in dimethyl sulfoxide (5 ml). The mixture was mechanically stirred and, after 48 hours, DMSA coated nanoparticles precipitated as a black powder. The supernatant liquid was removed and the black powder washed with ethanol (5 x 10 ml) and redispersed in water. NaOH(aq) was then added until pH = 10. The resulting black, homogeneous dispersion was dialyzed for 3 days, filtered (0.22 μm) and the pH adjusted to 7.4.

Characterization of IONPs.

Particle size and shape were examined by transmission electron microscopy (TEM microscope JEOL JEM 1010 operating at 100 kV) (**Suppl. Fig. S3A**). Samples were prepared by placing one drop of a dilute suspension in hexane onto a carbon coated copper grid and leaving it to dry at room temperature. The size distributions was determined through visual analysis of ensembles of over 300 particles found in randomly selected areas of the enlarged micrographs, with Image tool software to obtain the mean size and standard deviation.

The hydrodynamic size (DHYD) was measured by dynamic light scattering (DLS) using a Zetasizer Nano ZS TM (Malvern Instruments). Samples were diluted in ultrapure water at pH 7. The energy source was a laser emitting a red light, and the angle between the sample and detector is 173°. DHYD in this work refers to the Z-average size (**Suppl. Fig. S3B**).

The percent weight of the organic coating was obtained by thermogravimetric/differential thermal analysis (TGA/DTA) carried out in a TA Instruments TGA 500 apparatus, with a heating rate of 10 °C/min in air atmosphere from room temperature to 1000 °C. For this analysis, aqueous samples were lyophilized to obtain the corresponding powders.

Magnetic characterization of the sample was carried out in a vibrating sample magnetometer (VSM; Lakeshore model 7410). Magnetization curves were recorded at 298 K by first saturating the sample in a field of 2 T. M_S was evaluated by extrapolating to infinite field the experimental results obtained in the high field range, where the magnetization linearly increases with 1/H. The picture shows the changes in the magnetization (M) of the sample with an applied magnetic field (H) at 298 K. Nanoparticles exhibit a superparamagnetic behaviour with no magnetization when H = 0. The saturation magnetization value is M_S = 61 emu/g.

After carrying out the CMI method, labelled cells ($5x10^4$) were fixed with PFA 4% for 20 min at room temperature. Afterwards, the cells were washed twice with PBS and resuspended in 200 ul 1% agarose (50ºC) in 96 well plates excluding air bubbles. The samples were then scanned and T1, T2 and T2* weighted images were adquired with a ((*Bruker Pharmascan 7 Tesla (16cm)).*

A.

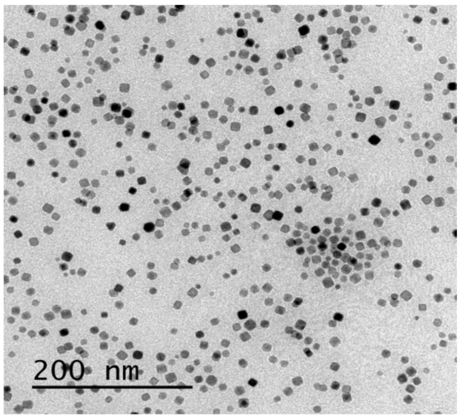

B.

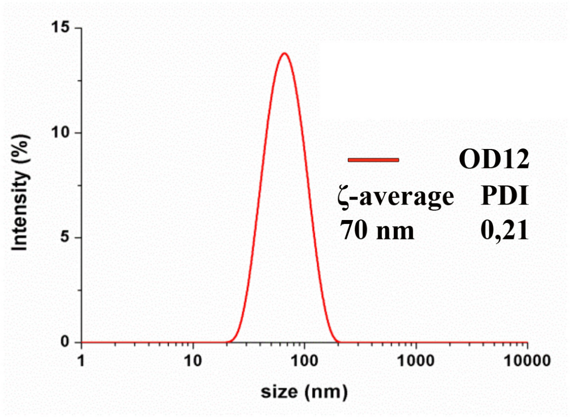

C.

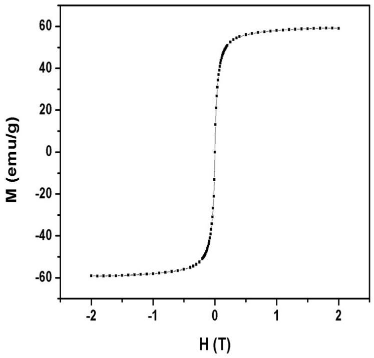

D.

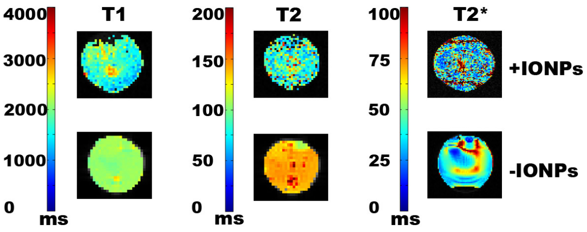

E.

MRI	R1	R2	R2*	R2/R1
Cells + IONPs	0,7	45,4	175,7	63,3
Cells - IONPs	0,3	8,9	8,8	26,6

Supplementary Figure S3: DMSA coated IONPs. A) Selected TEM micrograph of the IONPs used in this work; **B)** Hydrodynamic size distribution based on intensity, Z-average value and polidispersity (PDI) index of the IONPs (obtained from DLS measurements at 298 K). **C)** Changes in the magnetization (M) of the IONPs with an applied magnetic field (H) at 298 K. **D)** In vitro evaluation of iron labeling of U-251 cells by MRI. **E)** R values of cell phantoms with or without IONPs. For cell phantoms, U-251 cells were embedded in 1% agarose and MRI was performed using a high-field 7 tesla MR system ((*Bruker Pharmascan 7 Tesla (16cm)).*

Centrifugation-mediated internalization (CMI) video.

In order to supply with a visual representation of the methodology, we ordered the creation of *ad hoc* video to http://www.victorlavilla.com Angel Ayuso Sacido is the copyright holder of the video who grants permission to be published under an Open Access license (**Suppl. Video 1**)

Cell viability assays

Resazurin dye (Sigma-Aldrich) has been broadly used as a reliable indicator of cell viability in proliferation and cytotoxicity assay[12, 13]. Briefly, after cell treatment with IONPs (10, 25 and 50 μg Fe/ml) by DI or CMI methods, the medium was replaced with 600 ml of fresh medium containing 10% Resazurin dye and incubated at 37ºC for an additional 3 hours. The amount of reduced Resazurin was determined by measuring the absorbance of the sample using a UV-visible spectrophotometer (Sinergy H4 microplate reader) at wavelengths excitation of 570 nm and emission 600 nm. 600 ml of medium with 10% Resazurin dye was added to empty wells as a negative control. After absorbance measurement the medium was exchanged with fresh medium and the cells were incubated at 37ºC for 24 hours. Absorbance was measured using Resazurin dye as detailed before. For long-term cytotoxicity assay the process was repeated for 48, 72 and 96 hours. The viability of the cells was expressed as the percentage of absorption of treated cells in comparison with that of control cells and analyzed in triplicate (**Suppl. Fig. S8E, S10D, S11B** and **S12**).

Labeling efficiency of IONPs

After Prussian blue staining of U251 cells or hMSC, the labelling efficiency was determined by counting the number of Prussian blue stained and unstained labelled cells. Five random optic fields from each sample were taken by light microscope (Leica DMI3000B, Leica Microsystems, Germany). To estimate the percentage efficiency, the blue stained cell number was divided by the total cell number per field (**Fig. 2, 3C, 4B and 5B** and **Suppl Fig. S8B**).

Analysis of cell number versus IONPs concentration

To study the intracellular uptake efficiency of IONPs, $5x10^4$, $10x10^4$, $25x10^4$ and $50x10^4$ cells were divided in tubes of 15 ml and centrifuged at 1000 rpm for 5 minutes. The supernatant was removed and then, 500 μl of IONPs at concentrations of 25, 50,

125 and 250 µg Fe/ml were added over the cell pellets and centrifuged at 1500 rpm for 5 minutes. After that, the medium containing IONPs was exchanged with fresh medium without IONPs and the cells were plated at a density of $5x10^4$ cells per well in a 24 well plate. A plate of control cells was prepared in a similar manner without the addition of IONPs. Cells were incubated at 37ºC for 24 hours and staining with Prussian blue and counterstaining with neutral red as it has been described before. All experiments were carried out in triplicate (**Suppl. Fig. S9**).

Sample processing for Transmission Electron Microscopy (TEM).

For ultrastructural studies, U251 cells adhered to coverslips (both cells alone or cells with IONPs) were fixed in 2% paraformaldehyde and 2.5% glutaraldehyde for 1 hour and processed for embedding in the epoxy-resin EML-812 (TAAB laboratories, Berkshire, UK) as previously described elsewhere[14]. After washing with PBS and water, post-fixation of cells was done with 1% osmium tetroxide in PBS for 45 minutes. After extensive washing with water, samples were treated with 1% uranyl acetate in water for 45 minutes, washed again and dehydrated in increasing concentrations of ethanol (50, 75, 95 and 100%, 15 to 30 minutes each one). After 1 hour of incubation with a 1:1 mixture of ethanol and epoxy resin 812, cells were infiltrated with pure 812 epoxy resin at room temperature overnight. The polymerization of infiltrated samples was done in capsules for 2 days at 60ºC. Resin was detached from the coverslips by successive immersions in liquid nitrogen and hot water. Ultrathin (70-nm-thick) sections were obtained using a Leica EM UC6 ultramicrotome and a 35º-diamond knife (Diatome), transferred to formvar-coated EM buttonhole grids and stained with saturated uranyl acetate for 10 minutes and lead citrate for 3 minutes. Sections were visualized on a JEOL JEM 1011 electron microscope operating at 100 kV and equipped with a CCD camera (**Fig. 3A** and **Suppl. Fig. S7A**).

Supplementary references

1. Hinderliter, P.M. et al. ISDD: A computational model of particle sedimentation, diffusion and target cell dosimetry for in vitro toxicity studies. *Particle and fibre toxicology* **7**, 36 (2010).
2. Bird RB, S.W., Lightfoot EN Transport Phenomen. *John Wiley & Sons Inc* (1960).
3. Cho, E.C., Zhang, Q. & Xia, Y. The effect of sedimentation and diffusion on cellular uptake of gold nanoparticles. *Nat Nanotechnol* **6**, 385-391 (2011).

4. Fan, J. et al. Biological activity and magnetic resonance imaging of superparamagnetic iron oxide nanoparticles-labeled adipose-derived stem cells. *Stem Cell Res Ther* **4**, 44 (2013).
5. Richards, J.M. et al. In vivo mononuclear cell tracking using superparamagnetic particles of iron oxide: feasibility and safety in humans. *Circulation. Cardiovascular imaging* **5**, 509-517 (2012).
6. Soenen, S.J., Nuytten, N., De Meyer, S.F., De Smedt, S.C. & De Cuyper, M. High intracellular iron oxide nanoparticle concentrations affect cellular cytoskeleton and focal adhesion kinase-mediated signaling. *Small* **6**, 832-842 (2010).
7. Kustermann, E. et al. Efficient stem cell labeling for MRI studies. *Contrast Media Mol Imaging* **3**, 27-37 (2008).
8. Prentice P., C.A., Dholakia K., Prausnitz M., Campbell P. Membrane disruption by optically controlled microbubble cavitation. *Nature Physics* **1**, 107-110 (2005).
9. Prijic, S. et al. Increased cellular uptake of biocompatible superparamagnetic iron oxide nanoparticles into malignant cells by an external magnetic field. *The Journal of membrane biology* **236**, 167-179 (2010).
10. Park, J. et al. Ultra-large-scale syntheses of monodisperse nanocrystals. *Nat Mater* **3**, 891-895 (2004).
11. Salas G, C.C., Teran FJ , Miranda R, Serna CJ and Morales MP Controlled synthesis of uniform magnetite nanocrystals with high-quality properties for biomedical applications. *J. Mater. Chem* 21065-21075 (2012).
12. Ahmed, S.A., Gogal, R.M., Jr. & Walsh, J.E. A new rapid and simple non-radioactive assay to monitor and determine the proliferation of lymphocytes: an alternative to [3H]thymidine incorporation assay. *J Immunol Methods* **170**, 211-224 (1994).
13. Petrenko, Y.A., Gorokhova, N.A., Tkachova, E.N. & Petrenko, A.Y. The reduction of Alamar Blue by peripheral blood lymphocytes and isolated mitochondria. *Ukr Biokhim Zh* **77**, 100-105 (2005).
14. Chichon, F.J. et al. Cryo X-ray nano-tomography of vaccinia virus infected cells. *J Struct Biol* **177**, 202-211 (2011).

SUPLEMENTARY SECTION 3

SUPPLEMENTARY TABLES AND FIGURES

Supplementary Table S3: list of clinical studies involving IONPs internalization and *in vivo* cell tracking.

Clinical trial	Name	Phase
NCT00978562	Imaging Vascular Properties of Pediatric Brain Tumors Using Ferumoxytol and Gadolinium in a Single Imaging Session	recruiting participants
NCT00103038	MRI Using Ferumoxytol in Patients With Primary Brain Cancer or Brain Metastases	recruiting participants
NCT00660543	Magnetic Resonance (MR) Imaging Study Using Ferumoxytol to Assess Early Tumor Response in Patients With Glioblastoma Multiforme	Completed 2014
NCT00659126	Ferumoxytol and Gadolinium Magnetic Resonance Imaging (MRI) at 3 Tesla (T) and 7 Tesla (T) in Patients With Malignant Brain Tumors	recruiting participants
NCT00769093	Assessing Dynamic Magnetic Resonance (MR) Imaging in Patients With Recurrent High Grade Glioma Receiving Chemotherapy	recruiting participants
NCT00659776	MR, Histologic And EM Imaging Of Intravenous Ferumoxytol In Central Nervous System (CNS) Inflammation	recruiting participants
NCT00781872	Mesenchymal Stem Cells for the Treatment of MS	Completed 2008
NCT00972946	Cell Tracking Using Superparamagnetic Particles of Iron Oxide (SPIO) and Magnetic Resonance Imaging (MRI) - A Pilot Study	Completed 2009
NCT01169935	Tracking Inflammatory Cells Using Superparamagnetic Particles of Iron Oxide (SPIO) and Magnetic Resonance Imaging (MRI)	Completed 2012
NCT01127113	Inflammatory Cell Trafficking After Myocardial Infarction	Ongoing

Supplementary Table S4: Number of cells counted for each labeling efficiency assay.

Method	IONPs (ug/ml)	rpm	Time (min)	Analized Cells
CMI (Fig 4B)	10	1000	1	349
		1000	5	280
		1000	10	245
		1000	15	436
	25	1000	1	257
		1000	5	242
		1000	10	301
		1000	15	292
	50	1000	1	370
		1000	5	482
		1000	10	461
		1000	15	290
CMI (Fig 5B)	50	1000	1	133
		1000	5	139
		1000	10	184
		1000	15	133
	50	1000	1	145
		1000	5	152
		1000	10	138
		1000	15	163
DI (Sup.Fig 2)	25	0	1440	210
CMI (Sup. Fig 5B)	10	1000	5	280
		1250	5	327
		1500	5	425
	25	1000	5	242
		1250	5	578
		1500	5	434
	50	1000	5	579
		1250	5	324
		1500	5	397

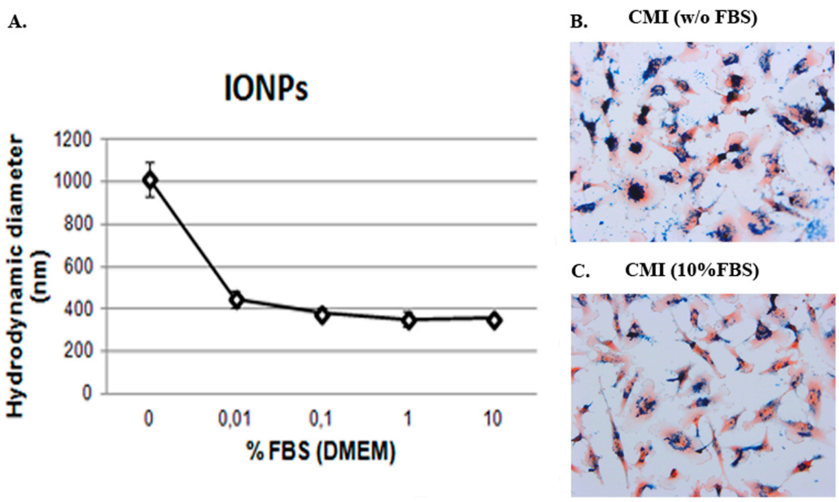

Supplementary Figure S4: FBS effect on the IONPs internalization by CMI method. A. Hydrodynamic diameter of the IONPs (obtained from DLS measurements at 298 K) with different concentrations of FBS**. B.** Prussian blue staining of cells following IONPs internalization by CMI for 5 minutes at 25 µg/ml and 1500 rpm without FBS. **C.** Prussian blue staining of cells following IONPs internalization by CMI for 5 minutes at 25 µg/ml and 1500 rpm with 10% FBS.

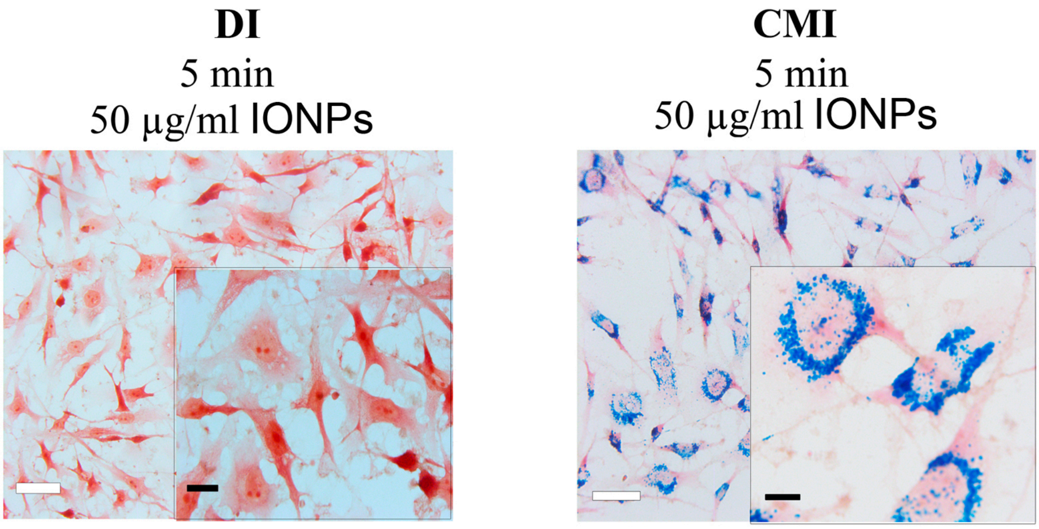

Supplementary Figure S5: Human tumour cells incubated for 5 min with IONPs by DI (left) and CMI (right) and stained with Prussian blue reaction for iron oxide detection.

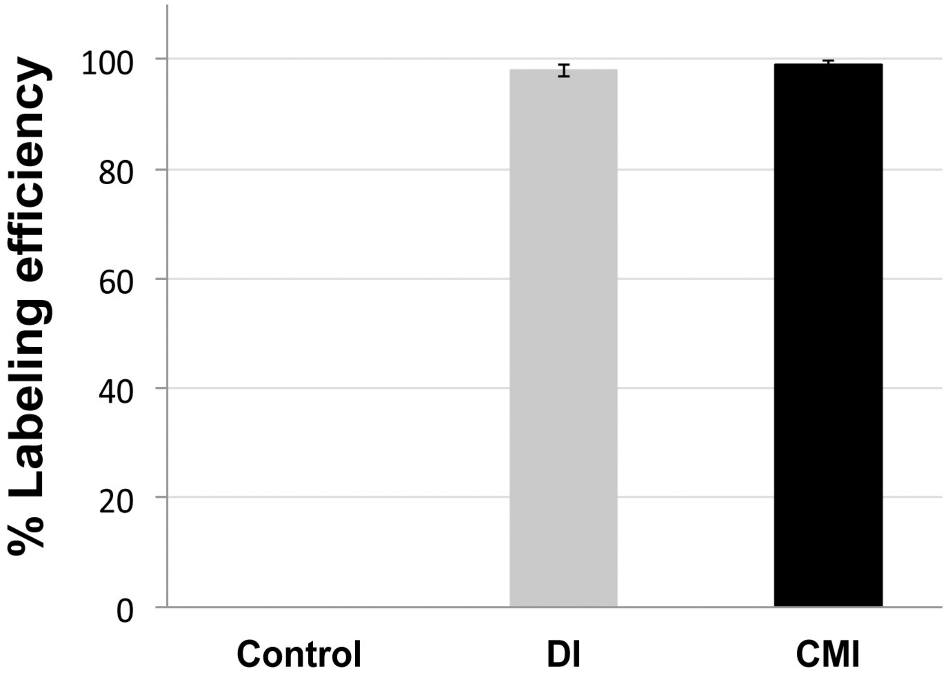

Supplementary Figure S6: Labelling efficiency of U251 with IONPs internalized by DI and CMI. The histogram represents the percentage of Prussian blue positive cells. Data are representative of 3 separated experiments. Error bars represent the standard deviation.

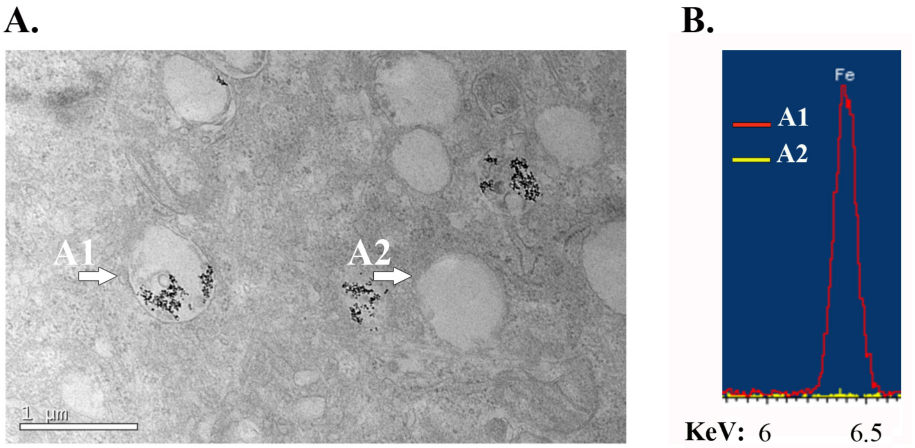

Supplementary Figure S7: TEM-EDX analysis of U251 cells after IONPs internalization by CMI. A) TEM image of U251 cells after IONPs internalization by CMI. The framed areas A1 and A2 indicate the vesicles selected for EDX analysis; **B**) EDX spectrum showing the elements composition of the selected vesicles (A1-A2). White arrows indicate iron composition within the vesicle A1. Note that the iron element was detected in vesicle A1 but not in vesicle A2, which had no visible IONPs.

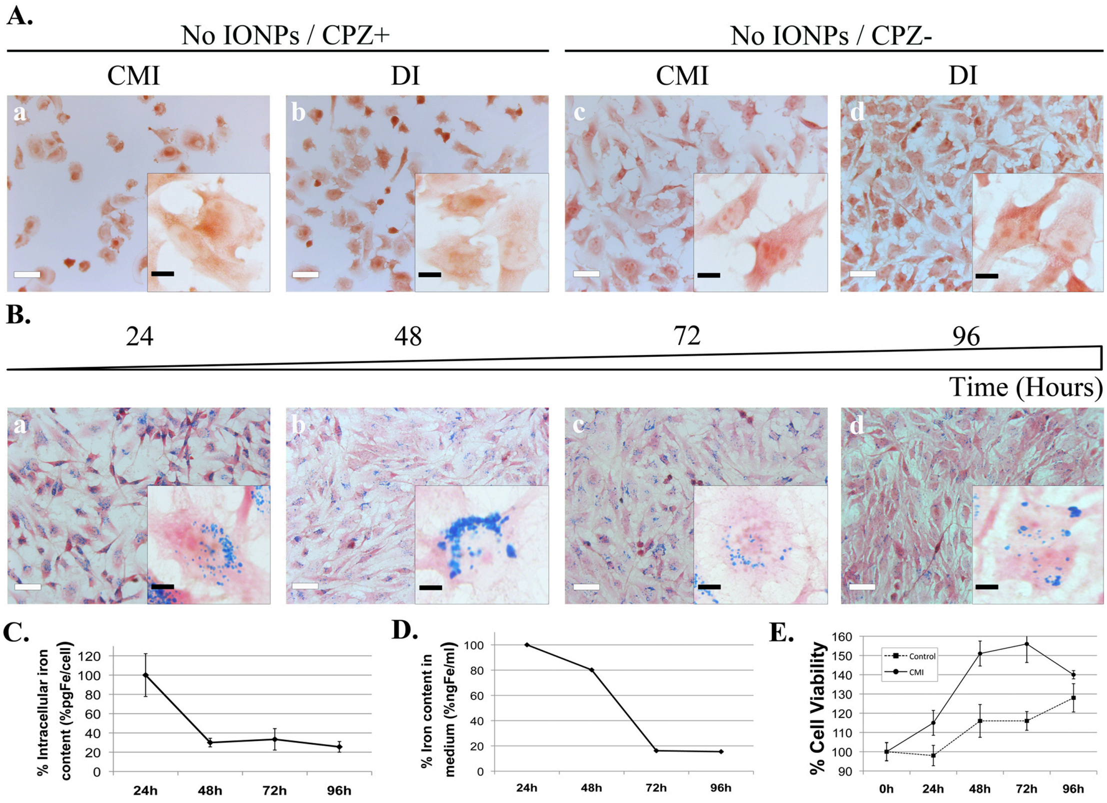

Supplementary Figure S8: IONPs internalization and release. A) Endocytosis inhibition by chlorpromazine (CPZ). Prussian blue staining of cells without IONPs after following DI (24 hours) or CMI (at 1500 rpm for 5 minutes) procedures in the presence or absence of the clathrin-inhibitor bychlorpromazine (CPZ) (10 µg/ml) for 5 hours. Data are representative of 3 separated experiments. Note: Blue stain density reflects the level of IONPs accumulation within cells. Scale bar: 40 µm and 10 µm; **B)** Prussian blue staining of U251 cells at different times (0, 24, 48, 72 and 96 hours) after internalization of IONPs (25 µg Fe/ml) by the CMI at 1500 rpm for 5 minutes method. Blue stain density reflects the level of IONPs accumulation within cells. Scale bar: 40 µm and 10 µm; **C)** Intracellular iron content measured by ICP; **D)** Iron content in the culture media measured by ICP; **E)** Cell viability determined by resazurin assay.

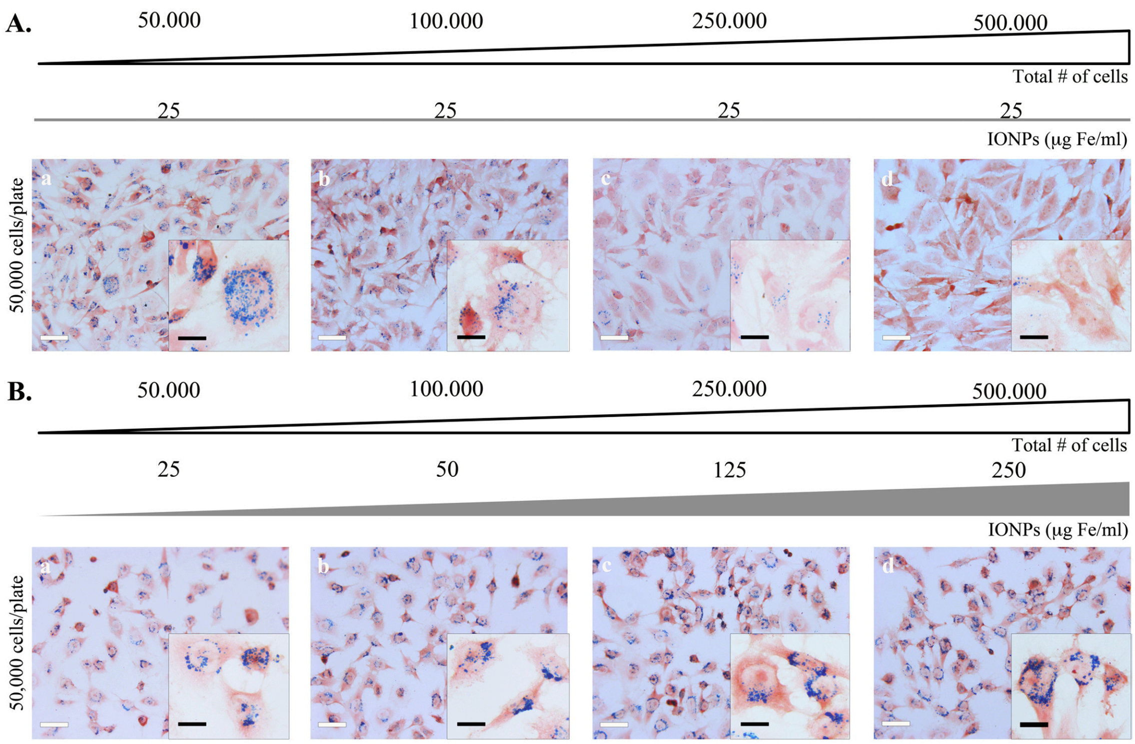

Supplementary Figure S9: The IONPs internalization efficiency depends on both the number of plated cells and the IONPs concentrations. A) IONPs were added at 50,000 (a), 100,000 (b), 250,000 (c) and 500,000 (d) cells per tube and CMI were performed at 25 μg/ml (a-d) at 1500 rpm for 5 minutes. Then, cells were plated at a density of 50,000 cells per well in 24 well/plates, incubated for 24 hours in normal medium without IONPs and staining with Prussian blue and counterstaining with neutral red; **B)** IONPs were added at 50,000 (a), 100,000 (b), 250,000 (c) and 500,000 (d) cells per tube and CMI were performed at 25 μg/ml (a), 50 μg/ml (b), 125 μg/ml (c) and 250 μg/ml (d) at 1500 rpm for 5 minutes. Then, cells were plated at a density of 50,000 cells per well in 24 well/plates, incubated for 24 hours in normal medium without IONPs and staining with Prussian blue and counterstaining with neutral red. Blue stain density reflects the IONPs accumulation within cells. Data are representative of total n=3 experiments. Scale bar: 40 μm and 10 μm.

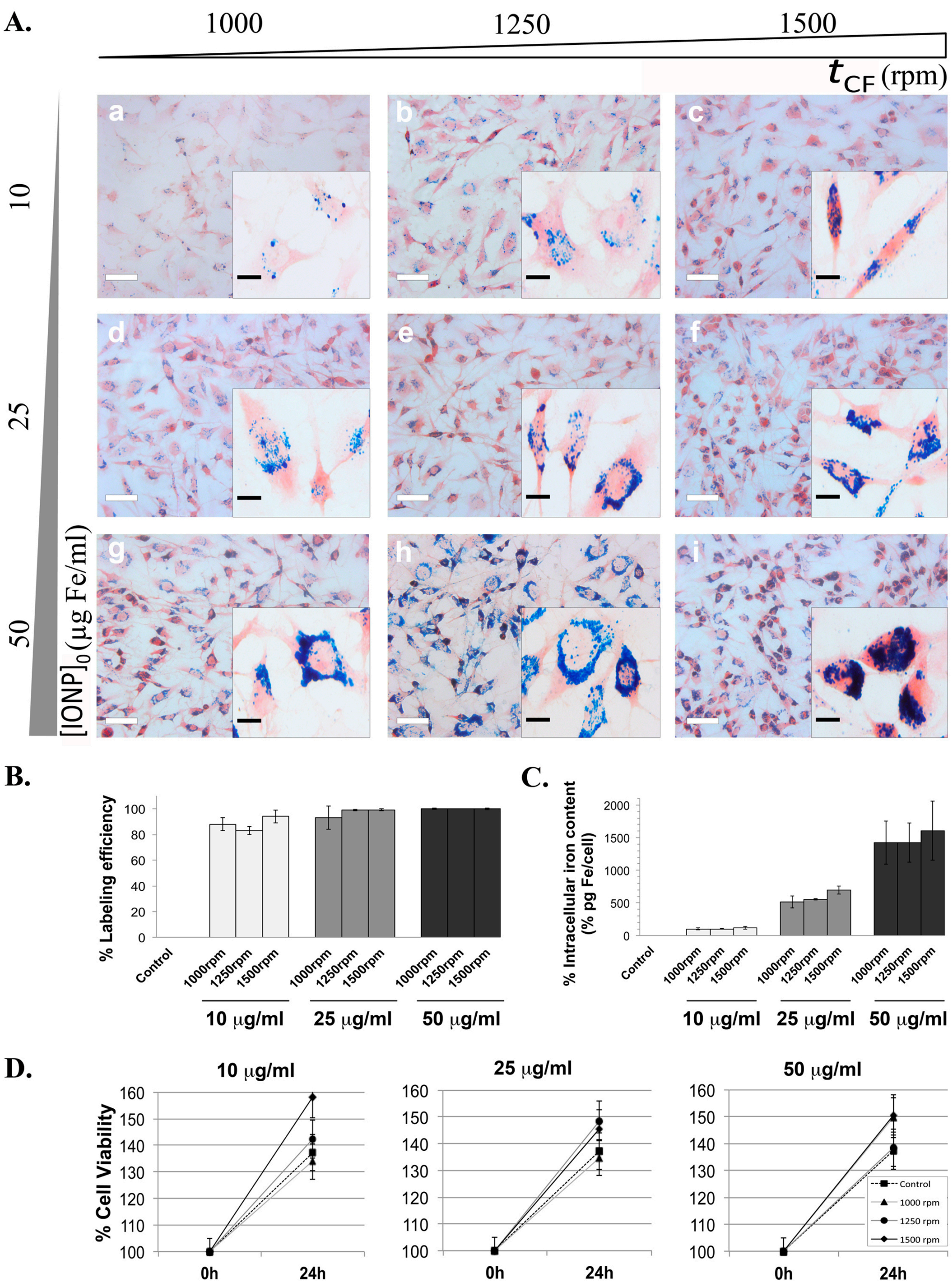

Supplementary Figure S10: Effect of the frequency of rotation in IONPs internalization into living cells by CMI. A) Prussian blue staining of cells following IONPs internalization by CMI for 5 minutes at 10, 25 and 50 µg/ml and different frequencies of rotation (1000, 1250 and 1500 rpm); **B)** Efficiency of cells labelling with IONPs; **C)** Intracellular iron content measured by ICP**; D)** Cell viability determined by resazurin assay. Blue stain density reflects the level of IONPs accumulation within the cells. Data are representative of 3 separated experiments. Scale bar: 40 µm and 10 µm. Error bars represent the standard deviation.

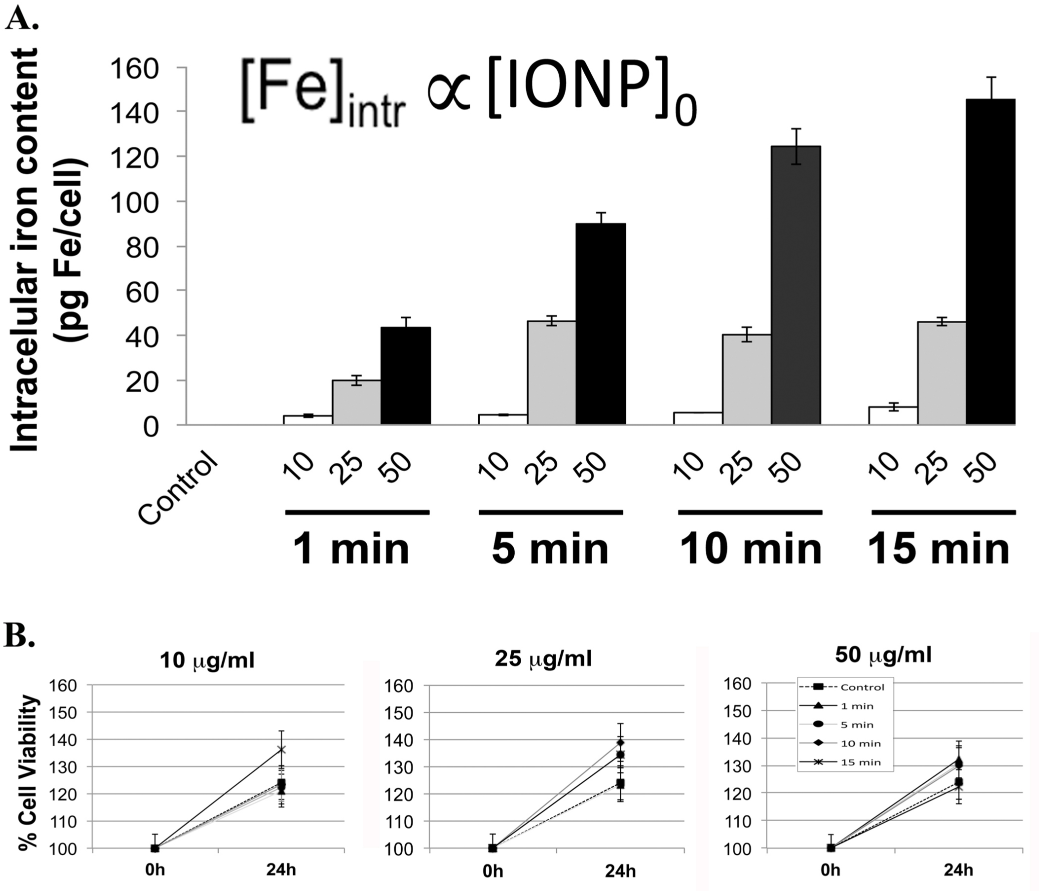

Supplementary Figure S11: Intracellular iron concentration and U251 viability assay. A) IONP internalization by CMI at 1000 rpm and different initial IONPs concentrations (10, 25 and 50 µg/ml) and Centrifugal Force application times (1, 5, 10 and 15 minutes). The histogram represents the intracellular Fe (pg Fe/ cell) for all different sceneries assayed; **B)** Cell viability after the Internalization of IONPs into U251 cells by CMI performed at 10, 25 and 50 µg/ml of initial IONPs concentration for 1, 5, 10 and 15 minutes of centrifugation time and 1000 rpm. Data are representative of total n=3 experiments. Error bars represent the standard deviation.

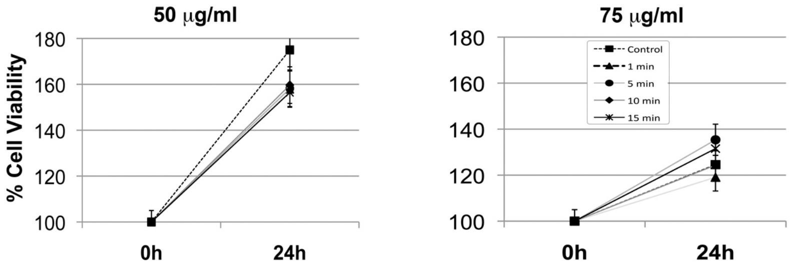

Supplementary Figure S12: hMSCs viability assay determined by resazurin assay. Cell viability after the internalization of IONPs into hMSCs by CMI performed at 50 μg/ml and 75 μg/ml of initial IONPs concentration for 1, 5, 10 and 15 minutes of centrifugation time and 1000 rpm. Data are representative of total n=3 experiments. Error bars represent the standard deviation.

Supplementary Video:

CMI video to provide for a visual representation of the methodology. The creation of *ad hoc* video was ordered to http://www.victorlavilla.com. Angel Ayuso Sacido is the copyright holder of the video who grants permission to be published under an Open Access license.
